# Eradication of an outbreak of vancomycin-resistant *Enterococcus* (VRE): the cost of a failure in the systematic screening

**DOI:** 10.1186/2047-2994-2-18

**Published:** 2013-06-06

**Authors:** Lélia Escaut, Samir Bouam, Marie Frank-Soltysiak, Eric Rudant, Faouzi Saliba, Najiby Kassis, Paul Presiozi, Daniel Vittecoq

**Affiliations:** 1 Department of infectious and tropical diseases, Bicêtre Hospital, Unité de Maladies Infectieuses et Tropicales, Hôpital Paul Brousse, 78, rue du Général Leclerc, Le Kremlin-Bicêtre 94270, France; 2 Department of hospital information, Paul Brousse Hospital, Villejuif, France; 3 Department of public health, Bicêtre Hospital, Le Kremlin-Bicêtre, France; 4 Laboratory of pharmacy, Paul Brousse Hospital, Villejuif, France; 5 Hepatobiliary centre, Paul Brousse Hospital, Villejuif, France; 6 Laboratory of microbiology, Paul Brousse Hospital, Villejuif, France; 7 ClinSearch, Bagneux, France

**Keywords:** Active surveillance, Outbreak, Cost, Vancomycin-resistant *Enterococcus*

## Abstract

**Background:**

Vancomycin-resistant enterococci (VRE) are still a concern in hospital units tending to seriously ill patients. However, the cost-effectiveness of active surveillance program to identify asymptomatically VRE colonized patient remains debatable. This work aims at evaluating the cost of a failure in the active surveillance of VRE that had resulted in an outbreak in a French University Hospital.

**Findings:**

A VRE outbreak was triggered by a failure in the systematic VRE screening in a medico-surgical ward specialised in liver transplantation as a patient was not tested for VRE. This failure was likely caused by the reduction of healthcare resource. The outbreak involved 13 patients. Colonized patients were grouped in a dedicated part of the infectious diseases unit and tended by a dedicated staff. Transmission was halted within two months after discovery of the index case.

The direct cost of the outbreak was assessed as the cost of staffing, disposable materials, hygiene procedures, and surveillance cultures.

The loss of income from spare isolation beds was computed by difference with the same period in the preceding year. Payments were drawn from the hospital database. The direct cost of the outbreak (2008 Euros) was €60 524 and the loss of income reached €110 915.

**Conclusions:**

Despite this failure, the rapid eradication of the VRE outbreak was a consequence of the rapid isolation of colonized patient. Yet, eradicating even a limited outbreak requires substantial efforts and resources. This underlines that special attention has to be paid to strictly adhere to active surveillance program.

## Introduction

Vancomycin-resistant enterococci (VRE) are a still growing concern in hospital units tending to seriously ill patients. VRE are easily transmitted via healthcare workers’ hands or clothes, or via contaminated instruments or environment [1]. The risk of VRE colonization resulting in systemic infection is substantially increased in immunosuppressed subjects, such as cancer patients or transplant recipients [2], and these infections are difficult to treat. Strategies to prevent VRE transmission include the use of barrier precaution, the isolation of colonized patients and active surveillance cultures to identify asymptomatically colonized patient. The effectiveness of active surveillance cultures that are cumulative expensive and resource intensive, in reducing VRE transmission remains controversial [3]. However, the non-compliance with active surveillance programs may outweigh the added expense of the program itself.

In this setting, this work evaluated the cost of a failure in the active surveillance of VRE that had resulted in an outbreak in a French University Hospital.

## Methods

### Setting

Paul Brousse hospital is a 716-bed (215 acute care beds) university hospital in Paris area. The hepato-biliary centre (HBC) is a 96-bed medico-surgical department, including a 15-bed intensive care unit (ICU), mainly involved in liver transplantation. Non-ICU beds spread across two floors. One of these receives liver transplant candidates and recipients while the other is dedicated to digestive surgery. The hospital also features a 20-bed infectious and tropical disease unit (IDU) located in a different building. In the first floor of HBC, systematic VRE screening is performed at the time of admission. Indeed, about 15% of patients hospitalized are foreigners, coming from geographical regions such as Southern Europe where the highest rates of VRE associated nosocomial infection have been reported [4].

A patient with end stage liver cirrhosis was admitted to the HBC from a Portuguese hospital on 31 August 2008. As there was no room on the floor devoted to liver transplantation since some beds had been closed for the summer, he was assigned to the surgical floor. There, he was not tested for VRE and no specific precautions were implemented. He was later transferred to the hepatologic floor where he was found colonized with an enteric vanA-type vancomycin-resistant *Enterococcus faecium* ten days after his admission. Following this discovery, patients were cohorted in the HBC by VRE status: positive, contacts or unknown. Contact patients, defined as present in the same sector or tended by the same staff as the index patient, were tested for VRE colonization. A total of 294 patients in the IDU and the HBC were screened for VRE colonization though a six weeks period. Taking into account the time needed to collect samples and to obtain results, two weeks were initially necessary to detect the first three additional patients colonized with the same strain. Consequently, it was decided to isolate these patients in a dedicated part of the IDU (12 beds) to maintain the surgical and liver transplantation activities.

These patients were tended by a dedicated medical and nursing staff. Strict contact precautions, hand hygiene, and disinfection procedures were implemented as recommended by guidelines [1]. Surveillance cultures were performed weekly on colonized patients and VRE-negative contacts from the IDU and the HBC. VRE-colonized patients were treated with a five-day course of oral bacitracin-streptomycin. Antibiotic prescription was monitored in the HBC and IDU in order to avoid unnecessary selection of resistant pathogen strains.

### Assessments

The staffing costs were evaluated as the overtime of staff dedicated to VRE patients, including training of healthcare and housing staff as well as information to patients or patients’ families. Interim staff, recruited for the duration of the outbreak to tend to non-VRE patients was also taken into account. The cost of disposable material and hygiene procedures was evaluated by comparison with their cost in the pre-outbreak period. Surveillance cultures and typing of isolates were valued using the official tariff for these procedures. The loss of admissions from spare isolation beds was assessed as the difference in admission rate in the IDU between the outbreak period and the same period in the preceding year. It was multiplied by the average cost of stay in the IDU.

## Results

Overall, the VRE colonized 13 patients. In one patient, VRE was also recovered from biliary drainage fluid. Two of them could be discharged directly from the HBC, the remaining eleven were treated in the IDU. No new colonization occurred in the HBC after 21 October — less than two months after discovery of the index case — and no case was observed among VRE-negative patients from the IDU seven weeks after the index case admission. The isolation sector was cancelled out on 1st December, after 60 days functioning.

The median age of the colonized patients was 59 years and most of the patients (83%) were male. Their underlying conditions were the following: hepatocellular carcinoma (n=7), uncompensated liver cirrhosis (n=9), liver transplantation (n=3), chronic viral hepatitis (n=4), HIV infection (n=1), cholangiocarcinoma (n=1) and gastric carcinoma (n=1). Six patients died from their underlying disease or from other infections. None of these deaths was related to the VRE. All the patients were successfully decontaminated as shown by three consecutive negative weekly cultures.

The median length of stay in hospital was 59 days (range, 22–77 days) whereas it was 29 days (range, 4–68 days) for the six patients that were in intensive care unit. In most patients, the length of stay was determined by the underlying condition or complications thereof. Yet, for one patient, transfer to an aftercare facility was delayed because of VRE colonization.

Liver transplantation activity, in the HBC was not impaired by the outbreak: 138 transplantations were performed in 2007, 130 in 2008 and 116 in 2009.

Hospital costs related to the management of VRE colonization in these patients are summarized in Table 1. The cost of infection control measures was four-fold higher than that of surveillance cultures performed weekly on colonized patients and VRE-negative contacts from the IDU and the HBC. These data also show that the direct cost of barrier precautions was modest.

**Table 1:** **Cost of the eradication of a vancomycin-resistant *Enterococcus faecium *(VRE) outbreak in severely ill colonized patients (n = 13 patients, 16 stays)**

**Resources**	Cost, 2008 Euros*
Infection control measures (A)	60 524
Staffing (overtime plus interim staff)	5 719
Gowns, gloves, single use materials, hydroalcoholic solutions, disinfection procedures	14 538
Screening and surveillance cultures, typing of isolates	15 125
Antibiotics	25 142
Loss of income from spare isolation beds† (B)	110 915
VRE-related hospital loss (A + B)	171 439

*One Euro was approximately equivalent to 1.30 US Dollar or 0.90 UK Pound.

†Loss of 33 admissions, compared to the same period in the preceding year.

## Discussion

This report indicates that eradicating even a limited outbreak requires substantial efforts and resources. Here, the outbreak was triggered by a failure in the systematic VRE screening. Indeed, the index patient was a candidate for liver transplantation. Owing to the increasing risk of VRE infection in transplant recipients, this patient should have been screened for VRE even if he was admitted in a non-hepatobiliary ward. Furthermore, the index patient came from Portugal where the prevalence of VRE among clinical Enterococcus faecium is approximately 25% [4]. Thus, this patient belonged to a population “at risk” for VRE colonization. Altogether, this suggests that the active surveillance program has failed. Such failure has been previously reported in liver transplant ICUs where active surveillance cultures were performed only on approximately 50% of the patients within 24h of admission [5]. In this study [5], the failure has been imputed to staff workload. In our study, same reason may have caused the nonfulfillment of systematic VRE screening. Altogether, this underlines that the implementation of the active surveillance program could have some limitations in the real life setting.

Despite this failure, the time taken to eradicate the VRE outbreak was rapid as it was less than two months. Indeed, experiences suggest than any delay in appropriate measures strengthens the threat of endemicity [6]. In this study, the involvement of the infection disease unit and hospital management provided the necessary leadership to enforce strict control measures. Consistently, no new colonization occurred once colonized patients were identified and transferred to infectious disease unit. This highlight that the isolation of colonized patient should not be delayed.

The cost of these measures that include the staffing, the barrier precautions and contact isolation far exceeded the cost of active surveillance cultures. However, one might wonder whether resort to such expensive measure was mandatory. Indeed, nosocomial pathogen outbreaks often trigger a debate between advocates of strict control measures and those who favor a less resource-intensive attitude, preserving usual clinical activity [7]. The Western Australian (WA) experience has convincingly shown that enhanced infection control practices are able to prevent transition from a large hospital outbreak to endemicity [8]. An often-debated measure is isolation, as opposed to in situ barrier precautions. In many hospitals, isolation sectors have to be set up by subtracting beds to routine clinical activity, which often results in a loss of income for the hospital since beds are then reserved for newly colonized or infected patients [6,7]. The case for isolation is not fully substantiated [9]. Simple cohorting may be sufficient where adequate architectural conditions are met (single-bed rooms, separate nurse station) and trained staff is present. Such conditions are often met in infectious disease units. Finally, the appeal of a resource-sparing infection control policy should be balanced against the likely and lasting cost of endemicity [10]. Estimating the cost-benefit of the eradication of a hospital outbreak is difficult as it depends on several factors, notably the delay before the next outbreak. However, Montecalvo *et al.*[10] showed that even in an endemic setting strict infection control measures may yield back up to 2.70 dollars for each dollar spent.

## Conclusion

Our report both underlines the burdensome and costly consequence of the non-compliance with active surveillance programs and the benefice of this program as the outbreak had been halted owing to the implementation of the VRE screening. This further suggests that beliefs related to the potential cost of VRE active surveillance programs should be balanced with the cost of the eradication of VRE outbreak. Thus, in the view of reducing health care cost, effort has to be made to strictly adhere to active surveillance program.

We acknowledge that the data presented here are somewhat anecdotal. They bear only on a limited outbreak, in a single clinical setting, within the framework of the French healthcare system. However, the failure resulting in this outbreak and caused by reduced resource in the summertime may likely be happened in the future. Indeed, the healthcare resource may be reduced due to the economic crisis. In this setting, our study warns that the improper implementation of active surveillance cultures related to lack of resource might increase VRE transmission and the associated expenses.

## Competing interest

The authors declare that they have no conflict of interest concerning this paper.

## Authors’ contributions

LE, DV and PP conceived the study and drafted the manuscript. SB, MFS, ER, FS and NK contributed to the acquisition, analysis and interpretation of the data, and revised the manuscript. All authors read and approved the final manuscript.

## Past presentation of the results

A partial report of these data has been presented as a poster at “Dixièmes Journées Nationales d’Infectiologie”, 10-12 June 2009, Lyon, France.
